# Immune Infiltration-Related Signature Predicts Risk Stratification and Immunotherapy Efficacy in Grade II and III Gliomas

**DOI:** 10.3389/fcell.2021.756005

**Published:** 2021-11-05

**Authors:** Cong Luo, Zhixiong Liu, Wenrui Ye, Fangkun Liu

**Affiliations:** ^1^Department of Urology, Xiangya Hospital, Central South University, Changsha, China; ^2^Department of Neurosurgery, Xiangya Hospital, Central South University, Changsha, China

**Keywords:** glioma, immune infiltration, prognosis, immunotherapy, function

## Abstract

**Background:** Tumor microenvironment, especially infiltrating immune cell, is crucial for solid tumors including glioma. However, the hub genes as well as their effects on patient prognosis and immunotherapy efficacy remain obscure.

**Methods:** We employed a total of 952 lower grade glioma (LGG) patients from The Cancer Genome Atlas (TCGA) and Chinese Glioma Genome Atlas (CGGA) databases, and 24 samples in our hospital for subsequent analyses. Abundances of immune infiltrates were evaluated using CIBERSORT and ImmuCellAI. Their correlations with prognosis were assessed by log-rank test. Immune infiltration-related hub genes were obtained from overlapped differential expressed genes (DEGs) in various subsets of survival-related immune cell types. The risk signature was constructed by Least Absolute Shrinkage and Selection Operator (LASSO) Cox regression analysis. The functional analyses were estimated by GVSA and Gene Set Enrichment Analysis (GSEA) algorithms. And protein–protein interaction enrichment analysis was carried out with the Metascape database integrating STRING, BioGrid, OmniPath, and InWeb_IM.

**Results:** Among the 21 infiltrates, the abundances of five immune infiltrates were correlated with overall survival (OS) in LGG patients. Higher abundances of naïve CD4+ T cells (*p* = 0.002), activated mast cells (*p* = 0.015), and monocytes (*p* = 0.014) were correlated with better prognosis, while higher abundances of resting memory CD4+ T cells (*p* = 0.015) and M1 macrophages (*p* = 0.020) correlated with poorer OS. We finally obtained 44 hub genes and constructed an immune infiltration-related signature (IIRS). The IIRS correlates with clinicopathological characteristics and exhibited potential power in predicting the immunotherapy efficacy. The IRRS correlates with cancer related pathways, especially “epithelial-mesenchymal transition (EMT),” and cytotoxic T lymphocytes.

**Conclusion:** Our study constructed and validated a novel signature for risk stratification and prediction of immunotherapy response in grade II and III gliomas, which was closely associated with glioma immune microenvironment and could serve as a promising prognostic biomarker for glioma patients.

## Introduction

Gliomas are the most common primary tumors of the central nervous system that arise from the intrinsic constituent cells of the brain (Sanai et al., 2005). They have historically been classified on the basis of their microscopic and immunohistochemical resemblance and have been graded according to histological features indicative of biological aggressiveness.

Genome-wide molecular-profiling studies have revealed comprehensive genomic landscapes for major types of gliomas (Suzuki et al., 2015). These developments have identified novel biomarkers for improved tumor classification and promising therapeutic targets. Predictive biomarkers recognized and used in clinics mainly included isocitrate dehydrogenase (*IDH*) mutation, the discovery of which constituted a key breakthrough in the understanding of WHO grade II/III gliomas (Yan et al., 2009). Besides, the presence of O6-methylguanine-DNA methyltransferase (MGMT) promoter methylation is predictive of response to temozolomide-based chemotherapy in patients with *IDH*-wild-type glioma (Wick et al., 2012). Furthermore, 1p/19q codeletion is suggested as a predictive marker of benefit from upfront combined radiotherapy and chemotherapy in two phase III trials (Bent et al., 2013; Cairncross et al., 2013). Novel pathogenesis-based treatments targeting oncogenic signaling pathways such as *BRAF* mutation (Robinson et al., 2014), epidermal growth factor receptor (EGFR) amplification (Phillips et al., 2016), and fibroblast growth factor receptor (FGFR)-TACC fusion demonstrate a potential for lower grade gliomas (LGG) elimination (Stefano et al., 2015). Despite a deconstruction at molecular level that furthered our understanding of tumorigenesis and personalized therapy, a certain LGG population acquired resistant to these targeted therapies.

For solid tumors, the tumor microenvironment, composed of extracellular matrix, stromal cells, and immune cells, plays a crucial role in the initiation and progression of cancer. The infiltrating immune cells there are very vital as they were associated with patient prognosis in various cancers (Yang et al., 2019; Huang et al., 2020; Zhang et al., 2020, 2021). Exploration of the glioma microenvironment will provide a better understanding of the occurrence and development of glioma. It is, however, worth noting that gliomas are not considered highly immunogenic since mutational loads are typically low, and gliomas are characterized by profound immunosuppression mediated by immune-inhibitory factors (Nduom et al., 2015). Therefore, we hope to decode the unique immune microenvironment and identify novel biomarkers to overcome immunosuppression, exploit antitumor immune responses, and guide individualized treatments.

We herein conducted a comprehensive analysis based on two independent cohorts, plus our own samples to explore the profile of infiltrating immune cells in gliomas in order to better understand its biological functions there. Furthermore, a risk signature based on immune infiltration (IIRS) was constructed to predict the prognosis of patients diagnosed with LGGs. Multifaceted performance of the IIRS was also examined to reveal its superior predictive ability for response to immunotherapy.

## Materials and Methods

### Data Extraction

All transcriptomic and clinical characteristics of enrolled samples were extracted from The Cancer Genome Atlas (TCGA) and Chinese Glioma Genome Atlas (CGGA) databases (Brat et al., 2015; Zhao et al., 2021). A total of 952 primary LGG samples with detailed clinical information were enrolled in our study, in which 508 samples extracted from the TCGA database were defined as the training set; 444 samples extracted from the CGGA database were defined as the validation set. Normal or glioblastoma (GBM, grade IV glioma) samples were excluded.

### Immune Infiltration and Survival Analysis in Lower Grade Gliomas

CIBERSORT algorithm was employed for evaluating the percentage of 21 human hematopoietic cell phenotypes, including seven T cell types, naïve and memory B cells, plasma cells, natural killer cells, and myeloid subsets (Newman et al., 2015). The associations between infiltrating abundance and overall survival (OS) in TCGA LGG cohort were evaluated using univariate Cox analysis, and the survival curves were correspondingly established by Kaplan–Meier analysis.

### Construction and Validation of the Immune Infiltration-Related Signature

Considering that the abundance of these infiltrating cells was mostly low, we used the mean abundance to divide the entire LGG population into high- and low-infiltrating groups for each infiltrate. For the immune infiltrates that were significantly correlated to the outcomes of LGG patients, we conducted a differential expression analysis using the R package “limma” and obtained fold change and *p*-value for each gene. Subsequently, for those infiltrating cells that were detrimental to patient prognosis, we selected genes ranked in the top 500 by fold change; whereas for those with a beneficial effect on patient prognosis, we selected genes whose fold change ranked in the bottom 500. Finally, we got five gene sets with 500 gene in each one.

Hub genes are derived from the intersection of the above gene sets, which were screened by using univariate Cox regression analysis. Thereafter, we used the R package “glmnet” to conduct Least Absolute Shrinkage and Selection Operator (LASSO) Cox regression analysis (with the penalty parameter estimated by 10-fold cross-validation), we developed an immune infiltration-related signature (IIRS) for the LGG patients. The risk score calculating formula is:


$$R\,i\,s\,k\,s\,c\,o\,r\,e=\sum_{i=1}^{n}\left(\beta_{i}\times x_{i}\right)$$

where “*n*” means the number of genes included in the model, “β_*i*_” means the LASSO coefficients, “*xi*” is the expression level of each model gene.

Risk scores were subsequently computed for all patients included in our study. For both cohorts, the patients were divided into high- and low-risk groups according to the median risk score. Then risk plots, scatter diagrams, heatmaps, survival curves, and time-dependent receiver operating characteristic curves (ROC) were plotted using the R package ‘‘ggplot2.’’ The relationships between risk signature and survival as well as other clinicopathological characteristics were also assessed. We calculated the correlations among the signature genes, as well as the correlations between individual gene and OS and progression free survival (PFS) in LGG populations. A principal components analysis (PCA) analysis, based on Gene Expression Profiling Interactive Analysis (GEPIA2^1^) webtool (Tang et al., 2019), was performed to examine the resolving power of the IRRS.

### Sample Collection and RNA Sequencing

Twenty-four samples were collected and then immediately stored in liquid nitrogen. Total RNA was extracted from the tissues using TRIzol (Invitrogen, Carlsbad, CA, United States) following the instructions. The mRNA library was then constructed after quantification using NanoDrop and Agilent 2100 bioanalyzer (Thermo Fisher Scientific, MA, United States). Total RNA was purified and fragmented into small pieces for cDNA synthetization. The cDNA fragments were further amplified by polymerase chain reaction after incubating with A-tailing mix and RNA Adapter Index for end repair. The qualified double-stranded PCR products were then used to construct the final library. Eventually, the 24 qualified glioma samples were further sequenced on a BGISEQ-500 platform (BGI-Shenzhen, China). The gene expression levels were calculated using RSEM (v1.2.12). The sequencing and clinical data of these samples were summarized in Supplementary Table 1.

### Prediction of Therapy Efficacy and Drug Response

The efficacies of four therapies (radiotherapy, chemotherapy, targeted therapy, and immunotherapy) in high-risk and low-risk groups were evaluated. We used Tumor Immune Dysfunction and Exclusion (TIDE^2^) algorithm to assess the ability of the IIRS in predicating response to the immunotherapy.

Two resources for therapeutic biomarker discovery in cancer cells, including Cancer Therapeutics Response Portal (CTRP) and Genomics of Drug Sensitivity in Cancer (GDSC) (Yang et al., 2012; Rees et al., 2016), were employed to evaluate the relationship between drug sensitivity (IC50) and mRNA expression.

### Gene Set Variation Analysis, Cancer Related Pathway, and Infiltrating Immune Cells

The Gene Set Variation Analysis (GSVA) score represents the integrated level of the expression of model gene set. The GSVA score of each patient was calculated using R package “GSVA” (Hänzelmann et al., 2013).

Data of reverse phase protein array (RPPA), a high-throughput antibody-based technique with the procedures similar to that of western blots, were used to calculate pathway activity score of 10 cancer related pathways, including TSC/mTOR, RTK, RAS/MAPK, PI3K/AKT, Hormone ER, Hormone AR, EMT, DNA Damage Response, Cell Cycle, Apoptosis pathways.

The infiltrates of 24 immune cells were evaluated through Immune Cell Abundance Identifier (ImmuCellAI) (Miao et al., 2020).

### Gene Set Enrichment Analysis, Mutational Profiles, and Protein–Protein Interaction

We calculated the degree to which the inputted gene set is overrepresented at the top or bottom of all genes ranked by gene expression fold change between high- and low-risk groups. The GSEA calculation was performed based on R package ‘‘fgsea.’’ The mutational profiles of model genes were assessed in cBioPortal website.^3^

For the differential expressed genes (DEGs) between high- and low-risk groups, protein–protein interaction enrichment analysis has been carried out with the Metascape databases integrating STRING (Szklarczyk et al., 2019), BioGrid (Stark et al., 2006), OmniPath (Türei et al., 2016), and InWeb_IM (Li et al., 2017). The resultant network contains the subset of proteins that form physical interactions with at least one other member in the list. The Molecular Complex Detection (MCODE) algorithm (Bader and Hogue, 2003) has been applied to identify densely connected network components.

### Statistical Analysis

Kaplan–Meier curve and log-rank test were used to compare the survival between various subgroups. The Student’s *t*-test was used to compare the risk scores between pairs of subgroups based on the following clinicopathologic features: age at initial pathologic diagnosis (≤40 vs. > 40 years old), gender (male vs. female), WHO grade (II vs. III), histological type (astrocytoma, oligoastrocytoma, and oligodendroglioma), and *IDH1* status (mutant vs. wild-type). Wilcoxon test and Kruskal–Wallis test were used for comparison between two groups, and for comparison among more than two groups, respectively. *p* < 0.05 was the significance threshold in most analyses. The statistical analyses were achieved by using R language (version 4.0.3).

## Results

### The Abundances of Five Immune Infiltrates Were Correlated With Overall Survival in Lower Grade Glioma Patients

**FIGURE 1:**
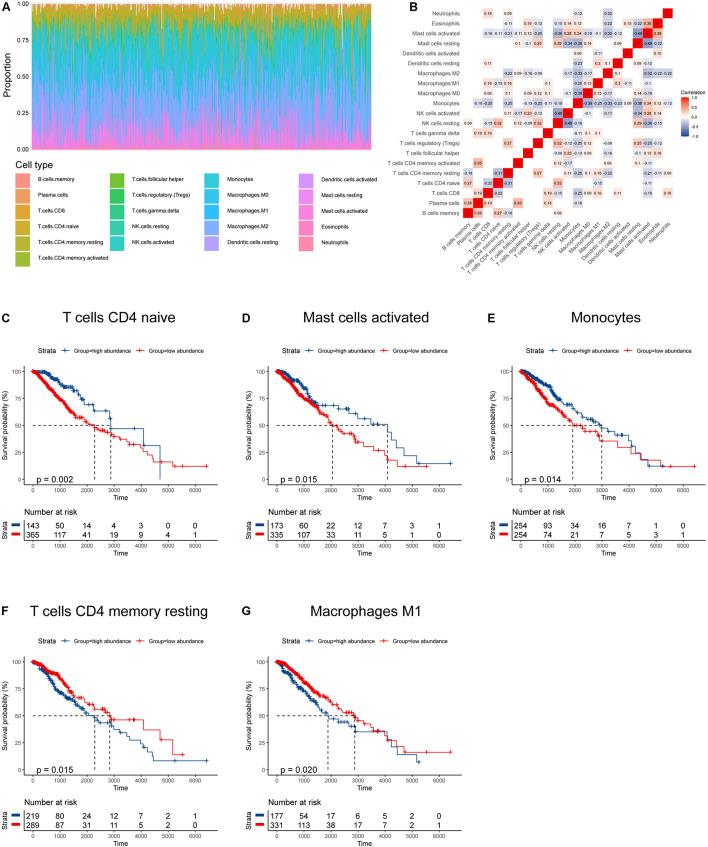
Immune infiltration profile and its relationship to survival in gliomas. **(A)** Proportions of various immune infiltrates. **(B)** The correlations among various immune infiltrates (non-significant values were omitted). **(C–G)** Kaplan–Meier survival curves based on immune infiltrates that were significantly related to patient prognosis.

We obtained data from two databases (TCGA, *n* = 508; CGGA, *n* = 444) to evaluate the abundances of immune infiltrates in LGGs. The characteristics of patients in the two cohorts were summarized in Table 1. The distribution and correlations of immune infiltrates in the TCGA cohort was displayed as Figures 1A,B. Among the 21 infiltrates, the abundances of five immune infiltrates were correlated with OS in grade II and III glioma patients (Figures 1C–G). Specifically, higher abundances of naïve CD4+ T cells (*p* = 0.002), activated mast cells (*p* = 0.015), and monocytes (*p* = 0.014) were related to better prognosis, while higher abundances of resting memory CD4+ T cells (*p* = 0.015) and M1 macrophages (*p* = 0.020) correlated with poorer OS.

**TABLE 1:** Detailed characteristics of included patients.

**Characteristics**	**TCGA (*n* = 508)**		**CGGA (*n* = 444)**	
	**N**	**%**	**N**	**%**
**Age (year)**				
≤40	251	49.41	229	51.58
>40	257	50.59	214	48.20
NA	0	0.00	1	0.23
**Gender**				
Female	282	55.51	193	43.47
Male	226	44.49	251	56.53
**Grade**				
II	246	48.43	189	42.57
III	262	51.57	255	57.43
**Histological type**				
A	128	25.20	271	61.04
OA	188	37.01	30	6.76
O	192	37.80	142	31.98
NA	0	0.00	1	0.23
***IDH1* status**				
Wild-type	34	6.69	96	21.62
Mutant	91	17.91	307	69.14
NA	383	75.39	41	9.23
**Radiation therapy**				
With	142	27.95	315	70.95
Without	119	23.43	102	22.97
NA	247	48.62	27	6.08
**Chemotherapy**				
With	277	54.53	285	64.19
Without	229	45.08	132	29.73
NA	2	0.39	27	6.08

*A, astrocytoma; IDH1, isocitrate dehydrogenase-1; NA, not available; O, oligodendroglioma; OA, oligoastrocytoma.*

### Identification of Candidate Genes and Construction of the Risk Signature

**FIGURE 2:**
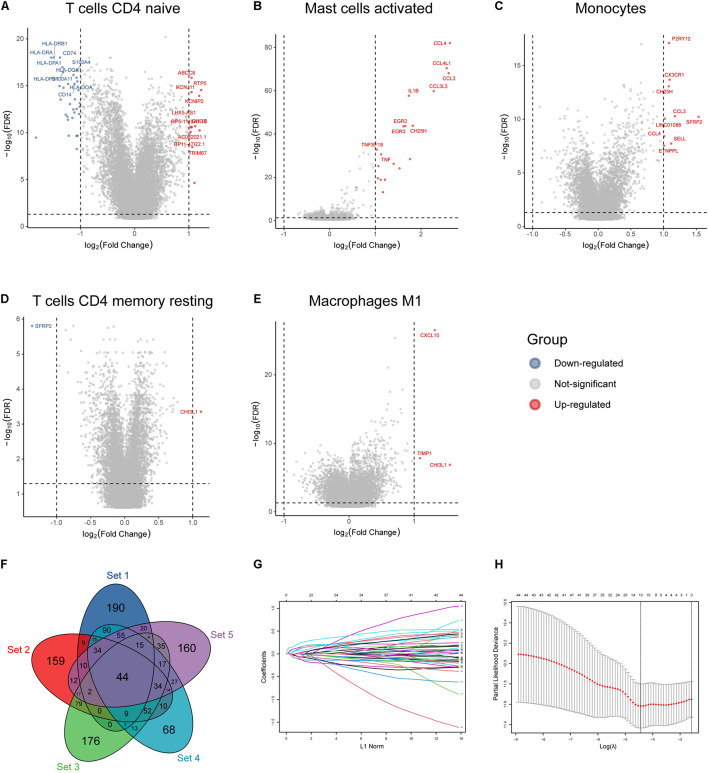
Identification of immune infiltration-related hub genes and LASSO analysis. **(A–E)** Volcano plot of DEGs based on the mean abundances of survival-related infiltrates in glioma. **(F)** Forty-four hub genes identified by overlapping the five gene sets. **(G)** LASSO coefficient profiles of the 44 hub genes. Each curve represents a coefficient, and the *x*-axis represents the regularization penalty parameter. As λ changes, a coefficient that becomes non-zero enters the LASSO regression model. **(H)** Cross-validation to select the optimal tuning parameter (λ). The left dotted vertical line crosses over the optimal log λ, which corresponds to the minimum value for multivariate Cox modeling.

According to the mean value of abundances of the five immune infiltrates, DEGs were obtained, respectively (Figures 2A–E). We profiled 500 candidate genes in these five groups, respectively. And 44 hub genes were finally obtained when overlapping the five sets containing 2,500 genes (Figure 2F). All 44 genes were significantly associated with patient outcome, and 10 genes were profiled by LASSO regression analysis to construct the IIRS (Figures 2G,H). Detailed descriptions, LASSO coefficients, and hazard ratios for all model genes are summarized in Table 2. And we exhibited the detailed expression levels as well as prognostic curves of these genes in Supplementary Figure 1. Briefly, all ten genes except *ACTN1* were differentially expressed between tumor and normal tissues. Among the remaining nine genes, *FABP* and *PLAT* were decreased in tumor tissues, while the others exhibited the opposite distribution (Supplementary Figure 1A). Regarding survival, these model genes were risk factors for OS, PFS, and disease specific survival (DSS) in patients with LGG (Supplementary Figure 1B). And the specific Kaplan–Meier survival curves (for OS) were displayed in Supplementary Figure 1C.

**TABLE 2:** Detailed information of model genes.

**Model genes**	**Description**	**Coefficient**	***p*-Value**	**HR**	**Lower 95% CI**	**Higher 95% CI**
*IGFBP2*	Insulin-like growth factor binding protein 2	−0.09331651	9.05E-29	1.63	1.50	1.78
*PLAT*	Plasminogen activator	0.10371271	1.34E-19	1.61	1.45	1.78
*TOP2A*	Topoisomerase (DNA) II alpha	0.07377614	4.86E-12	1.50	1.34	1.69
*ACTN1*	Actinin, alpha 1	0.1394003	3.01E-10	1.54	1.35	1.76
*LGALS3*	Lectin, galactoside-binding, soluble, 3	−0.06051677	2.33E-11	1.59	1.38	1.81
*BGN*	Biglycan	−0.11792178	7.56E-12	1.57	1.38	1.79
*FMOD*	Fibromodulin	0.12075655	2.49E-15	1.42	1.30	1.54
*FABP5*	Fatty acid binding protein 5	−0.05734956	2.18E-19	1.74	1.54	1.96
*BCAT1*	Branched chain amino-acid transaminase 1	0.22682052	1.14E-16	1.80	1.57	2.07
*COL1A2*	Collagen, type I, alpha 2	0.02175721	2.18E-09	1.49	1.31	1.70

*CI, confidence interval; HR, hazard ratio.*

### External and Subgroup Validation Demonstrates Stability of the Immune Infiltration-Related Signature

**FIGURE 3:**
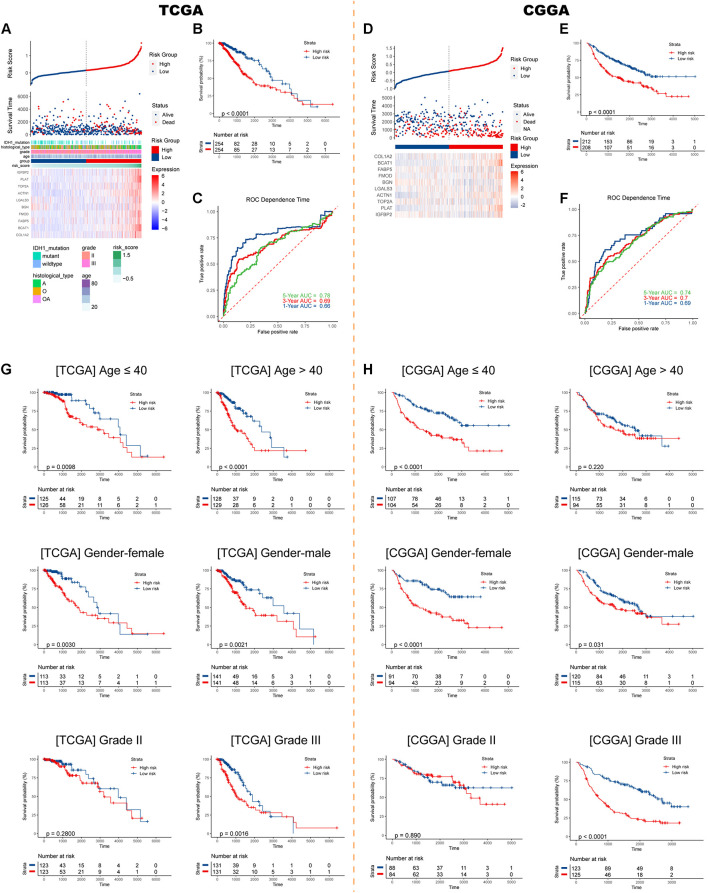
Construction and validation of the IIRS. **(A)** Risk score, survival status, and expression pattern of model genes in each patient in the training cohort. **(B)** Kaplan–Meier analysis of patients in the high- and low-risk groups. **(C)** Time-dependent ROC analysis of the IIRS in predicting prognosis. **(D–F)** Validation of the IIRS in the CGGA cohort. **(G,H)** Kaplan–Meier analysis of patients in the high- and low-risk groups in stratified subgroups in the TCGA and CGGA cohorts.

Risk plots, survival distributions, and model gene expressions were plotted in Figure 3A. Kaplan–Meier survival curve indicated that LGG patients with higher risk scores had significantly worse outcomes in the training set (*p* < 0.0001, Figure 3B). The time-dependent ROC curve demonstrated a promising ability of the model to predict OS in the training cohort (1-year AUC = 0.66, 3-year AUC = 0.69, 5-year AUC = 0.78; Figure 3C). The results were similar in the external CGGA cohort (Figure 3D). Higher risk scores also indicated poorer OS (*p* < 0.0001, Figure 3E). The risk model retained stable and high predication ability (1-year AUC = 0.69, 3-year AUC = 0.70, 5-year AUC = 0.74; Figure 3F). These results showed that the IIRS had a robust and stable OS-predictive ability for LGG patients. Furthermore, we performed a stratification analysis and found that the risk model maintained the ability to predict OS in most subgroups in both cohorts (Figures 3G,H).

### The IRRS Correlates With Clinicopathological Characteristics and Predicts Immunotherapy Efficacy

**FIGURE 4:**
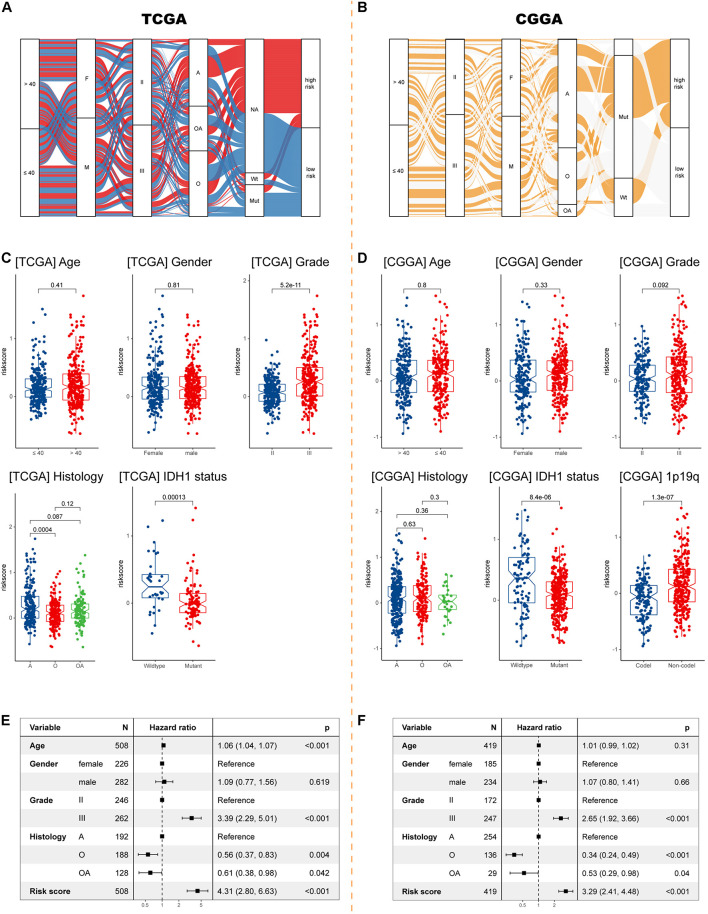
Exploration the relationship between clinicopathologic characteristics and the IIRS. **(A,B)** Sankey diagrams exhibiting the distribution of clinicopathologic characteristics in high- and low-risk groups in the two cohorts. **(C,D)** The risk score in different subgroups stratified by age, gender, grade, histological type, *IDH1* status, and 1p/19q codeletion status in the TCGA and CGGA cohorts. **(E,F)** Cox analyses examining different variables for LGG patient survival in the TCGA and CGGA cohorts.

Sankey diagrams were displayed showing the distribution of risk scores and clinicopathologic characteristics among LGG patients (Figures 4A,B). In the TCGA cohort, LGG patients with higher WHO grade had higher risk scores, while the risk score was not associated with gender. Besides, an individual patient would have a higher risk score if he had a pathologic type of astrocytoma (Figure 4C). In the CGGA cohort, risk scores were higher in patients with 1p/19q codeletion (Figure 4D). Importantly, LGG patients with wild-type *IDH1* would have higher risk scores in both cohorts. Further Cox analyses showed that higher age, higher grade, pathological type (astrocytoma), and higher risk score were significantly associated with worse survival in both cohorts (Figures 4E,F).

**FIGURE 5:**
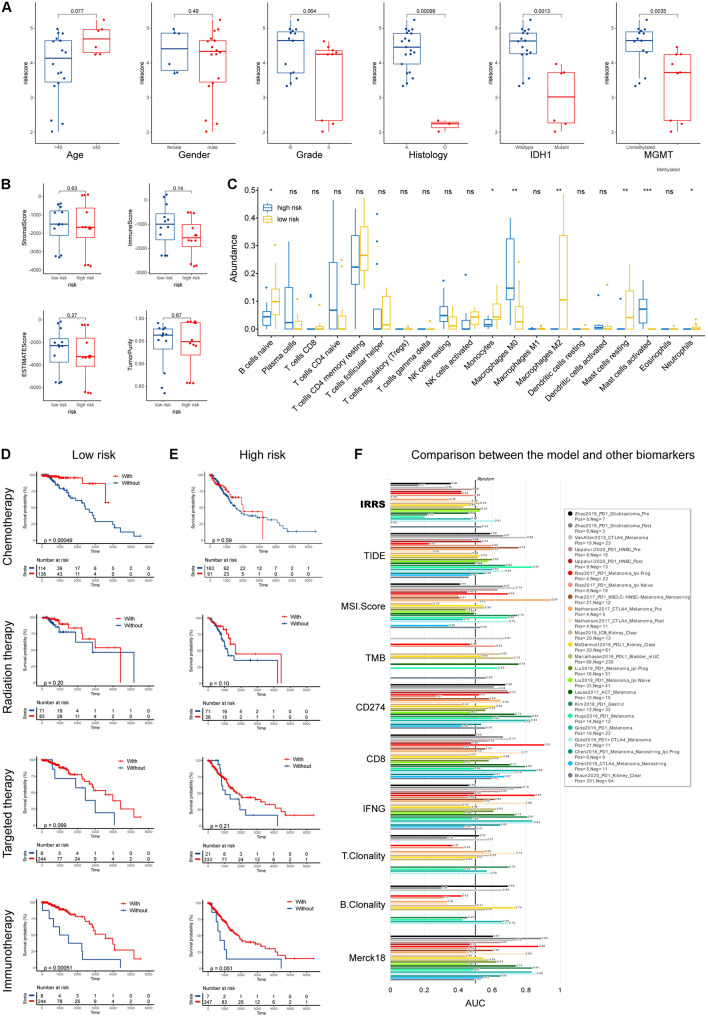
Validation using collected samples and prediction of therapy efficacy. **(A)** The risk score in different subgroups stratified by age, gender, grade, histological type, *IDH1* status, and MGMT status using own samples. **(B)** Stromal, immune, and ESTIMATE scores compared between high- and low-risk groups. **(C)** The abundances of various immune cells in the high- and low-risk groups. **(D,E)** The efficacies of chemotherapy, radiation therapy, targeted therapy, and immunotherapy in the low- and high-risk groups. **(F)** Comparison of the abilities in predicting the response to immunotherapy with recognized biomarkers or genes. *p < 0.05, **p < 0.01, ***p < 0.001, ns: not significant.

Using samples from Xiangya hospital, we found the consistent results that glioma patients with histological type of astrocytoma, wild-type *IDH1*, and unmethylated MGMT had higher risk scores (Figure 5A). Although no differences in tumor purity were observed between low- and high-risk groups (Figure 5B), we found that, in our samples, high risk patients had higher abundances of M0 macrophages and activated mast cells, and lower abundances of naïve B cells, monocytes, M2 macrophages, resting mast cells, and neutrophils (Figure 5C).

Furthermore, we investigated the efficacies of multiple treatments in low- and high- risk groups (Figures 5D,E). Chemotherapies demonstrated high efficacies in both groups, while radiation and targeted therapies did not improve patients’ outcome. Intriguingly, patients in the low-risk group uniquely responded well to immunotherapy. To better evaluate the potential of our IIRS in predicting patients’ responses to immunotherapies, we employed TIDE algorithm to compare our model with other published biomarkers in immunotherapy response prediction (Figure 5F). Compared with recognized signatures (or genes) including Merck18, TIDE score, microsatellite instability (MSI) score, tumor mutation burden (TMB), CD274, CD8, IFNG, T clonality, and B clonality, the IIRS showed robust ability in predicting the response to immunotherapies in patients with different cancers. Besides, the correlations between drug sensitivity and model gene mRNA expressions were exhibited in Supplementary Figures 2A,B.

### The IRRS Correlates With Cancer Related Pathways and Cytotoxic T Lymphocytes

**FIGURE 6:**
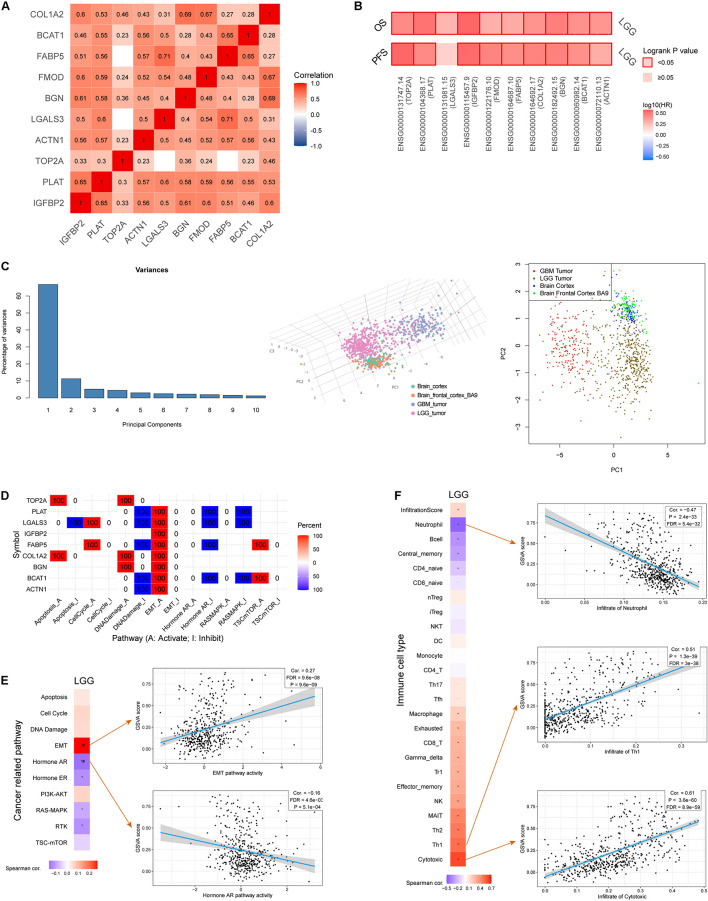
Comprehensive analysis of model genes in the IIRS. **(A)** Spearman correlations among the model genes. **(B)** Cox analyses exhibiting the relationships between the expressions of model genes and OS and PFS. **(C)** PCA analysis. **(D)** Involvement of individual model genes in cancer related pathways. **(E)** Correlation between GSVA score and cancer related pathways. **(F)** Correlation between GSVA score and immune cell types.

To better understand how the IRRS participates in oncologic processes, we analyzed our model both intrinsically and extrinsically. Strong and significant correlations existed among the model genes (Figure 6A), all of which were risk factors for the OS in the LGG cohort (Figure 6B). Besides, the expressions of all genes, except for *LGALS3*, were correlated with poorer PFS in patients with LGG (Figure 6B). The comprehensive investigation in the mutational profiles of model genes was displayed as Supplementary Figure 3, where we found BGN was most likely to mutate in LGGs. These mutations were significantly related to molecular status including *IDH*, 1p, and 19q. A subsequent PCA analysis suggested the IRRS could distinguish LGG from normal cerebral cortex tissues and glioblastomas (Figure 6C).

Next, we evaluated the involvement of individual genes in various cancer related pathways, and found that all genes participated in activated “epithelial-mesenchymal transition (EMT)” pathway while five of them were involved in inhibited “DNA damage” pathway (Figure 6D). Furthermore, GVSA score was significantly and positively correlated with EMT pathway (*r* = 0.27, *p* = 9.6e-9) while negatively correlated with “Hormone androgen/androgen receptor (AR)” pathway (*r* = −0.16, *p* = 5.1e-4) (Figure 6E).

We then focused on the association of the IRRS and multiple immune cell types (Figure 6F). Strong and positive associations were observed between GVSA scores and activated T lymphocytes including cytotoxic T lymphocytes (*r* = 0.61, *p* = 3.6e-60) and type 1 helper T cells (*r* = 0.51, *p* = 1.3e-39). In contrary, the GVSA score was strongly and negatively correlated with the abundance of neutrophil (*r* = −0.47, *p* = 2.4e-33).

### Functional Analysis Reveals Deep Involvement of Immune Infiltration-Related Signature in the Glioma Immune Microenvironment

**FIGURE 7:**
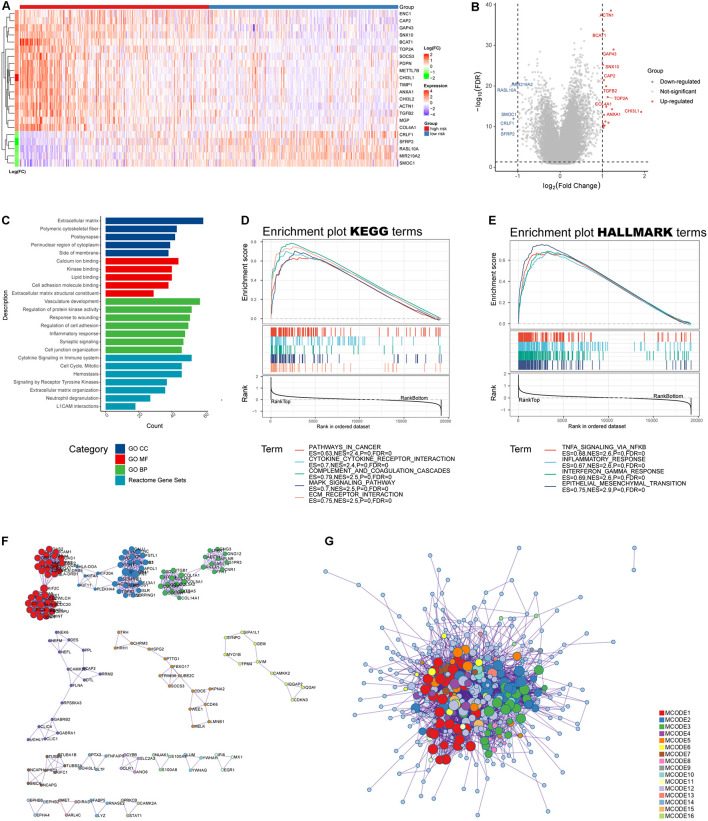
Functional analysis of the IRRS. **(A)** Heatmap of differential expressed genes between high- and low-risk groups. **(B)** Volcano plot of differential expressed genes (log_10_FDR < 0.05, | log_2_(fold change)| > 1). **(C)** GO and Reactome enrichment analysis. **(D,E)** GSEA analysis for KEGG and HALLMARK terms. **(F,G)** Protein–protein interaction displayed by enriched MCODE networks.

We obtained DEGs between low- and high-risk groups (Figure 7A), which were displayed as volcano plot (Figure 7B). Functional analysis indicated these DEGs were mainly located in extracellular matrix and were involved in binding functions such calcium ion, kinase, and cell adhesion molecule binding (Figure 7C). As for biological processes, they participated in responses to wounding and inflammatory responses (Figure 7D). Furthermore, the gene sets were mainly enriched in several Reactome pathways including “cytokine signaling in immune system,” “hemostasis,” and “neutrophil degranulation” (Figure 7E). To further capture the relationships between the terms, a subset of enriched terms has been selected and rendered as a network plot, where terms with a similarity above 0.3 are connected by edges. We select the terms with the best *p*-values from each of the 20 clusters and visualized the network as Supplementary Figures 2C,D (Shannon et al., 2003).

The MCODE networks identified for individual gene lists have been gathered and are shown in Figures 7F,G. Pathway and process enrichment analysis has been applied to each MCODE component independently, and the three best-scoring terms by *p*-value have been retained as the functional description of the corresponding components: (1) GO:0002399, MHC class II protein complex assembly (log_10_*p* = −15.7); (2) GO:0002503, peptide antigen assembly with MHC class II protein complex (log_10_*p* = −15.7); and (3) GO:0009611, response to wounding (log_10_*p* = −15.4).

## Discussion

Glioma is a common tumor in human central nervous system. Over the past decades, surgical section with radiotherapy and chemotherapy still represented the mainstream treatment against glioma. Due to the unique microenvironment, gliomas acquire immunosuppressive phenotypes and poorly response to established immunotherapies. Therefore, novel targets are in urgent need for the hope to predict response rate and improve patient outcome. We employed comprehensive bioinformatic analyses to build an IIRS, which helps clinicians to optimize the management of LGGs. We believe that the IRRS is a good predictor of outcomes in LGG, and targeting the model genes there will demonstrate encouraging efficacy in future preclinical and clinical practice.

Immune infiltration has gained widespread attention in the last decade, especially its unique involvement in malignant processes such as tumor progression and immunotherapy resistance, making it a promising target in tumor microenvironment (Hinshaw and Shevde, 2019). Previous studies have investigated the relationships between immune infiltration and prognosis in various cancer types. High abundance of M2 macrophages was reported to be related to poorer survival in patients with bladder cancers, while CD8+ T cells were related to improved prognosis in this cancer type (Zhang et al., 2020). In addition, CD4+ naive T cells, regulatory T cells, M2 macrophages, resting mast cells were identified as risk immune cells in digestive system cancers, while the abundances of naive B cells, CD8+ T cells, CD4 memory activated T cells, follicular helper T cells, and eosinophils were correlated with better relapse free survival (Yang et al., 2019). To the best our knowledge, this study is the first to comprehensively evaluate the effects of various immune infiltrates on glioma patients’ survival. We assessed the profile of immune infiltration, and found that naïve CD4+ T cells, activated mast cells, and monocytes were protective factors, while resting memory CD4+ T cells and M1 macrophages were risk factors for the prognosis of patients with grade II and III gliomas.

More recent studies have reported that myeloid cells and B cells in the meninges mainly originate from the calvaria bone marrow, rather than the peripheral circulation (Brioschi et al., 2021; Cugurra et al., 2021). Here, B cells mature and develop in the meninges rather than in the bone marrow as recognized (Brioschi et al., 2021). Immune cells may be directly transported through vessels present between the skull and dura. These findings collectively point to the fact that the brain, unlike peripheral organs, has a “self-sufficient” and relatively independent immune system. Further studies should be conducted to assess whether these cells elicit similar effects as we estimated. Meanwhile, studies should be conducted to examine whether these effects are mediated by cytotoxic T lymphocytes or helper T cells.

Forty-four hub genes were selected and used to establish the IIRS. The signature retained stability in different dataset and subgroups. For the model genes in the IIRS, previous studies had provided evidence for their effects in malignancies. Insulin like growth factor (IGF)-binding protein 2 (*IGFBP2*) is an IGF system regulator and a developmentally regulated gene. Accumulating evidence indicates that in solid tumors, *IGFBP2* is upregulated and promotes several key oncogenic processes, such as epithelial mesenchymal transition, cell migration, invasion, angiogenesis, stemness, transcriptional activation, and epigenetic programming through signaling, thus being a hub of oncogenic networks and a potential therapeutic target for cancer treatment (Li T. et al., 2020). Furthermore, in a study including 2447 glioma samples with gene expression profiles, *IGFBP2* was found to be involved in immunosuppressive activities and was an independent unfavorable prognostic biomarker (Cai et al., 2018). Actinin alpha 1 (*ACTN1*) has been identified as a glioma microenvironment-related gene with prognostic value in malignant gliomas (Li Y. et al., 2020). Moreover, lectin, galactoside-binding, soluble, 3 (*LGALS3)* is a poor prognostic factor in diffuse glioma (Hu et al., 2020), and it can promote therapeutic resistance there (Wang et al., 2019). Fibromodulin (*FMOD*) was upregulated in glioma and could promote glioma cell migration by inducing the formation of filamentous actin stress fibers. Both *FMOD* promoter methylation and transcript levels predict prognosis in gliomas (Mondal et al., 2017). Furthermore, fatty acid binding protein 5 (*FABP5*) was identified as one of the most enriched genes and its elevation revealed severe outcomes in malignant LGGs. And the malignant properties of LGGs were promoted by exogenous overexpression of *FABP5* through tumor necrosis factor α-dependent NF-κB signaling (Wang et al., 2021). Importantly, a high-quality study linked metabolism and tumors, where it found that gliomas expressed high levels of branched chain amino-acid transaminase 1 (*BCAT1*). Inhibition of *BCAT1* in glioma cell lines blocked glutamate excretion and resulted in decreased proliferation and invasiveness *in vitro*, as well as a significant decrease in tumor growth in a glioma xenograft model, indicating a central role of *BCAT1* in glioma pathogenesis (Tönjes et al., 2013). Additionally, collagen, type I, alpha 2 (*COL1A2*) was identified as a hub gene in glioma in several studies (Yin et al., 2021). These findings comprehensively supported our IRRS in glioma, as most model genes were elaborated to be involved in the initiation, progression, or treatment resistance in gliomas.

A key finding in our study is that glioma patients in the low-risk group exhibited a unique response to immunotherapy, as those who received immunotherapy had significantly improved survival compared with those who did not. Taken together with the TIDE algorithm showing the excellent predictive ability of our model for immunotherapy response, we conclude that the IIRS can reflect the sensitivity of LGGs to immunotherapy and recommend this model to guide clinical decisions.

There were several limitations in our study. First, the size of samples for validation was too small, thus the accuracy of the validation is open to question. Second, we included 10 genes in the IIRS, proposing a great challenge for experimental validation. Although we used patients from the CGGA database (validation set) to validate the results obtained from the TCGA database (training set) and showed good concordance, multicenter cohorts with large sample size and complete clinical data are still needed to elaborate our conclusions. Third, there is no LGG cohort in the TIDE database, so the prediction of response to immunotherapy in LGG needs further validation.

## Conclusion

In summary, our study establishes a model based on glioma immune infiltration profiles that accurately predicts patient prognosis and response to immunotherapy, with the expectation of aiding decision making in clinics.

## Data Availability Statement

The datasets presented in this study can be found in online repositories. The names of the repository/repositories and accession number(s) can be found below: NCBI SRA BioProject, accession no: PRJNA767573.

## Ethics Statement

The studies involving human participants were reviewed and approved by the Ethical Committee of Xiangya Hospital (No. 2017121019). The patients/participants provided their written informed consent to participate in this study.

## Author Contributions

WY performed the data analysis and interpreted the data and prepared the draft. FL collected the samples in our cohort and they were responsible for the subsequent RNA sequencing of them. CL performed the visualization. ZL and FL revised the manuscript. CL and FL designed the research and supervised all the work. All authors have read and approved the final manuscript, and agreed to be accountable for the content of the work.

## Conflict of Interest

The authors declare that the research was conducted in the absence of any commercial or financial relationships that could be construed as a potential conflict of interest.

## Publisher’s Note

All claims expressed in this article are solely those of the authors and do not necessarily represent those of their affiliated organizations, or those of the publisher, the editors and the reviewers. Any product that may be evaluated in this article, or claim that may be made by its manufacturer, is not guaranteed or endorsed by the publisher.

## Footnotes

1
http://gepia2.cancer-pku.cn

2
http://tide.dfci.harvard.edu

3
https://www.cbioportal.org

## Supplementary Material

Supplementary Figure 1 **(A)** Expression levels of model genes between tumor and normal tissues. **(B)** Survival map for OS, DSS, PFS, and disease free interval (DFI) with hazard ratios and cox *p*-values. **(C)** Kaplan–Meier survival curves of model genes.

Supplementary Figure 2 **(A,B)** Correlations between drug sensitivity and model gene expression in the CTRP and GDSC databases. **(C,D)** Networks of enrichment terms based on similarity with best p-values.

Supplementary Figure 3 Mutational profile of model genes. **(A)** Oncoprint mutation status, **(B)** lollipop charts, and **(C)** copy number variations of model genes. **(D)** Distinction of molecular features between mutant and wild-type groups (*p* < 0.01).
