# Supplementary figures and images for: Immune Infiltration-Related Signature Predicts Risk Stratification and Immunotherapy Efficacy in Grade II and III Gliomas
Source: Front Cell Dev Biol. 2021 Nov 5;9:756005. doi: 10.3389/fcell.2021.756005 (PMC8603377; doi:10.3389/fcell.2021.756005)

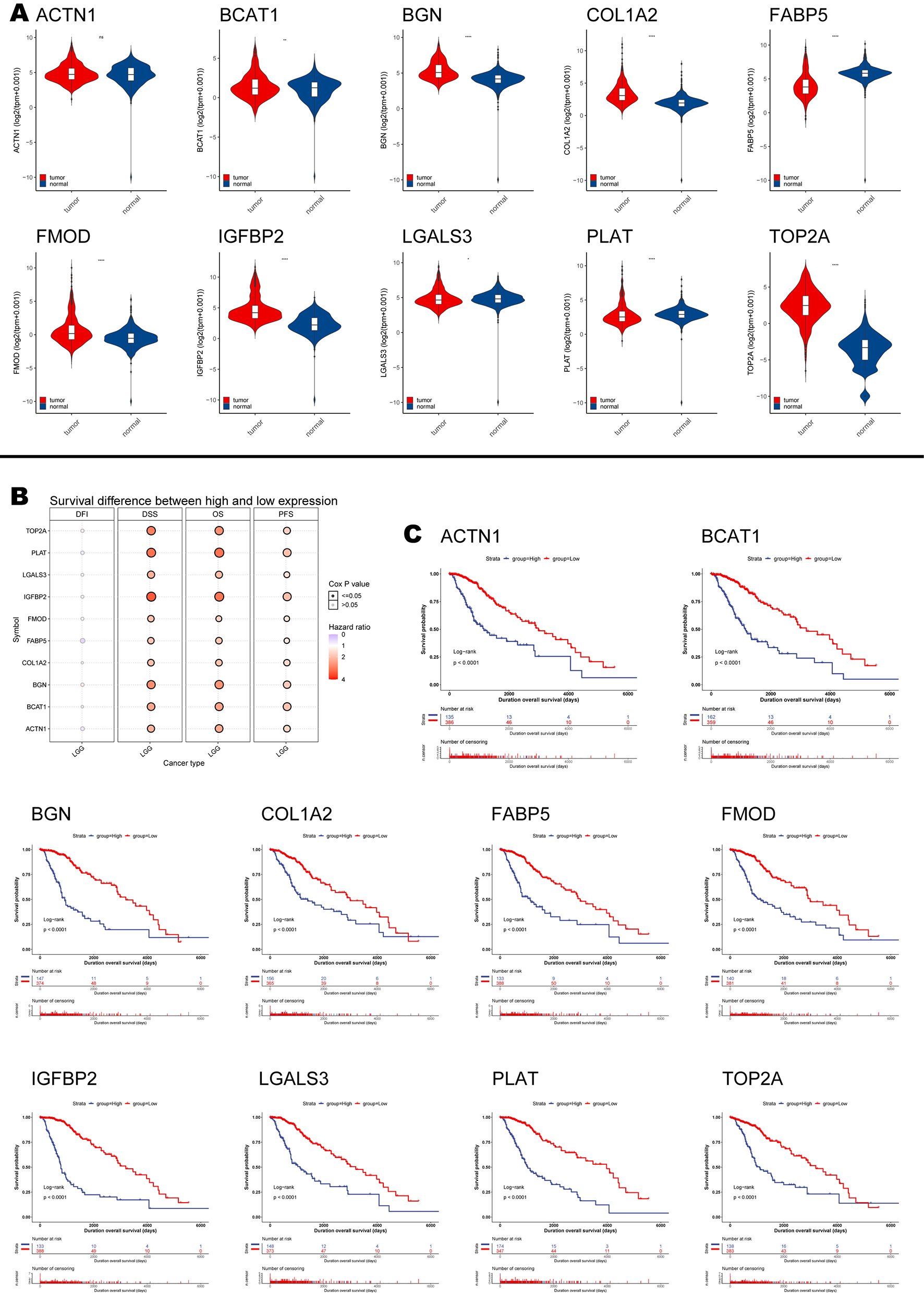

Supplement: Supplementary Figure 1 — (A) Expression levels of model genes between tumor and normal tissues. (B) Survival map for OS, DSS, PFS, and disease free interval (DFI) with hazard ratios and cox p-values. (C) Kaplan–Meier survival curves of model genes. [file Image_1.JPEG]

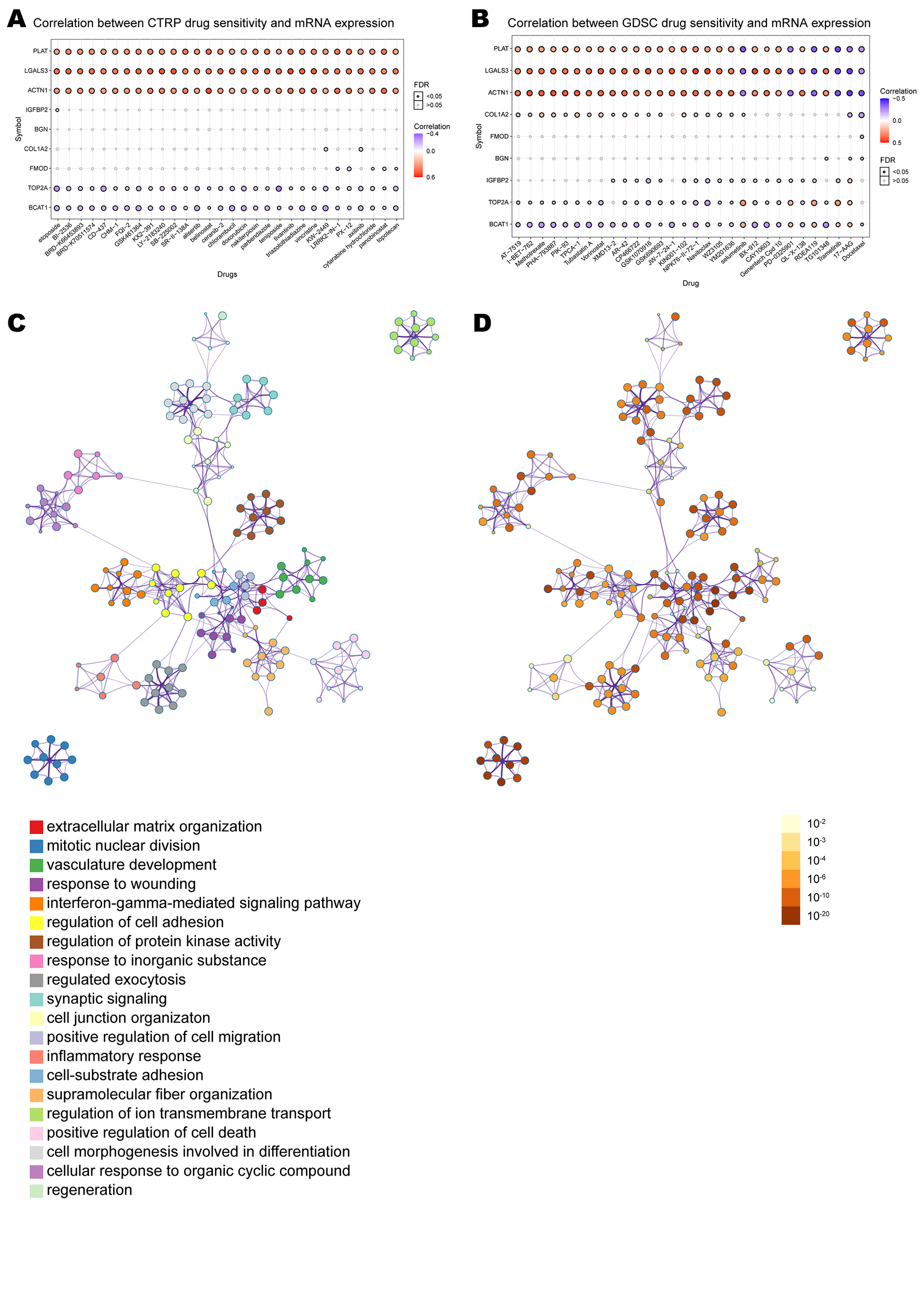

Supplement: Supplementary Figure 2 — (A,B) Correlations between drug sensitivity and model gene expression in the CTRP and GDSC databases. (C,D) Networks of enrichment terms based on similarity with best p-values. [file Image_2.JPEG]

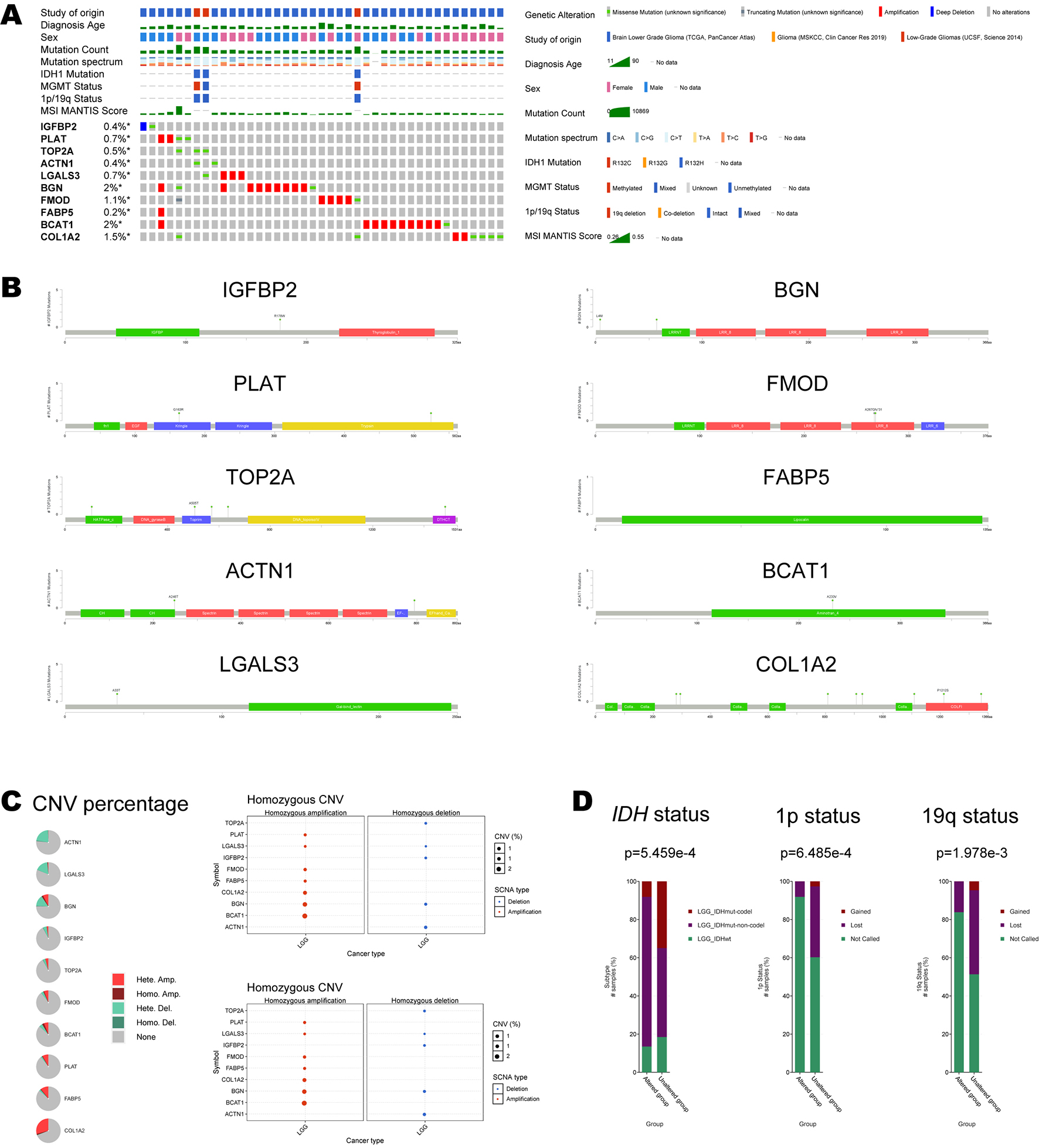

Supplement: Supplementary Figure 3 — Mutational profile of model genes. (A) Oncoprint mutation status, (B) lollipop charts, and (C) copy number variations of model genes. (D) Distinction of molecular features between mutant and wild-type groups (p < 0.01). [file Image_3.JPEG]
